# Innovative Microstructural Transformation upon CO_2_ Supercritical Conditions on Metal-Nucleobase Aerogel and Its Use as Effective Filler for HPLC Biomolecules Separation

**DOI:** 10.3390/nano12040675

**Published:** 2022-02-17

**Authors:** Noelia Maldonado, Garikoitz Beobide, Efraim Reyes, José Ignacio Martínez, Carlos J. Gómez-García, Oscar Castillo, Pilar Amo-Ochoa

**Affiliations:** 1 Department of Inorganic Chemistry, Autonomous University of Madrid, 28049 Madrid, Spain; noelia.maldonado@uam.es; 2 Department of Organic and Inorganic Chemistry, University of the Basque Country (UPV/EHU), 48080 Bilbao, Spain; garikoitz.beobide@ehu.eus (G.B.); efraim.reyes@ehu.eus (E.R.); oscar.castillo@ehu.eus (O.C.); 3 BC Materials, Basque Center for Materials, Applications and Nanostructures, UPV/EHU Science Park, 48940 Leioa, Spain; 4 Department of Nanostructures, Surfaces, Coatings and Molecular Astrophysics, Institute of Materials Science of Madrid (ICMM-CSIC), 28049 Madrid, Spain; joseignacio.martinez@icmm.cesic.es; 5 Departamento de Química Inorgánica, Universidad de Valencia, Dr. Moliner 50, 46100 Burjasot, Spain; carlos.gomez@uv.es; 6 Institute for Advanced Research in Chemistry at UAM (IAdChem), Universidad Autónoma de Madrid, 28049 Madrid, Spain

**Keywords:** metal–organic gels, metal–organic aerogels, analytical applications, coordination polymers

## Abstract

This work contributes to enlightening the opportunities of the anisotropic scheme of non-covalent interactions present in supramolecular materials. It provides a top-down approach based on their selective disruption that herein has been employed to process a conventional microcrystalline material to a nanofibrillar porous material. The developed bulk microcrystalline material contains uracil-1-propionic acid (UPrOH) nucleobase as a molecular recognition capable building block. Its crystal structure consists of discrete [Cu(UPrO)_2_ (4,4′-bipy)_2_ (H_2_ O)] (4,4′-bipy=4,4′-bipyridine) entities held together through a highly anisotropic scheme of non-covalent interactions in which strong hydrogen bonds involving coordinated water molecules provide 1D supramolecular chains interacting between them by weaker interactions. The sonication of this microcrystalline material and heating at 45 °C in acetic acid–methanol allows partial reversible solubilization/recrystallization processes that promote the cross-linking of particles into an interlocked platelet-like micro-particles metal–organic gel, but during CO_2_ supercritical drying, the microcrystalline particles undergo a complete morphological change towards highly anisotropic nanofibers. This unprecedented top-down microstructural conversion provides a nanofibrillar material bearing the same crystal structure but with a highly increased surface area. Its usefulness has been tested for HPLC separation purposes observing the expected nucleobase complementarity-based separation.

## 1. Introduction

Coordination complexes and polymers (CCs and CPs) are fascinating compounds formed by the combination of different building blocks through coordination bonds, mainly metal ions and organic molecules [1,2]. The building blocks, structure, and morphology mark the final properties of the obtained compounds [3,4,5,6,7]. In addition, the incorporation in the CPs or CCs of organic ligands with molecular recognition abilities allows the design of 3D networks to be based not only on coordination bonds but also using weaker supramolecular interactions such as hydrogen bonds or van der Waals [8,9,10,11]. The presence of anisotropic supramolecular interactions provides interesting strategies for incorporating new properties into these materials [12]. 

The study of morphological transformations in CPs is fascinating since a small structural change can significantly affect their physicochemical properties. For this reason, studies have been carried out on structural changes related to the variation in pH [13], the concentration of reagents [12], the presence of other components [14,15], the application of ultrasound [16,17], among others [18,19,20]. Additionally, the particle size of CCs can be reduced from micrometers to nanometers by modifying their synthetic conditions allowing new and exciting applications [21,22].

The fine-tuning of the synthetic conditions can even produce metal–organic gels (MOGs) in which these particles are interlinked into a meso/macroporous material [23,24]. In this regard, although increasingly understood, the gel’s formation mechanisms remain far from being settled knowledge as many factors can affect both their formation and stability [14,25,26,27,28,29,30,31]. Moreover, these new materials are linked to nanomaterials science since their obtaining implies mostly a bottom-up approach (to our knowledge, no precedents have been found for the preparation of metallogels via top-down approach) [32]. 

Light air-filled 3D microstructures, called metal–organic aerogels (MOAs), with attractive mechanical [25] or chemical responsiveness properties [33,34], can also be created through supercritical drying (CO_2_) of MOGs [35,36,37]. Indeed, by combining Cu(OAc)_2_, uracil-1-acetate (UAcO) and 4,4′-bipyridine (4,4′-bipy), we have recently designed a mono-dimensional CP composed of a double chain with a [Cu_2_(μ-CH_3_COO)_2_(μ-4,4′-bipy)_2_] core decorated with terminal UAcO residues. The control of the experimental conditions allows the preparation of the corresponding MOA, whose mesoporous microstructure is formed by entangled nanoribbons with a specific surface area of 21 m^2^ g^−1^ [22].

In this work, uracil-1-propionic acid (UPrOH) replaces the uracil-1-acetic acid to provide a new coordination compound of formula [Cu(UPrO)_2_(4,4′-bipy)_2_(H_2_O)] (CuUPrO), in which the coordination bond polymerization is avoided, and its crystal structure is completely sustained by an anisotropic network of non-covalent interactions. The particle morphology of the pulverulent bulk material consists of quasi-hexagonal micrometric platelets. The use of an unprecedented top-down approach with ultrasound, temperature, and small amounts of acetic acid produces its gelation (CuUPrO@MOG), retaining the morphology of the micrometric particles. The CO_2_ supercritical drying leads to the corresponding metal–organic aerogel (CuUPrO@MOA). Interestingly, this change gave rise to a rare microstructural transformation of the previously platelet micrometric particles into nanofibers with a very high aspect ratio while retaining the internal crystallographic structure. This phenomenon, as far as we know, is unprecedented. The new morphology of the particles observed in CuUPrO@MOA provides a larger specific surface area [22]. This fact would lead in principle to an enhanced interaction capability of this new material and a better separation performance than previously observed for CuUAcO@MOA [22]. However, the mechanical properties of these materials do not allow them to be directly incorporated in HPLC columns as they are unable to withstand the pressure gradient, and their porous microstructure collapses in a dense, compact form that blocks the flux along the column. To overcome this drawback, we employed a mechanical mixture of a small amount of CuUPrO@MOA with micrometric quartz particles (SiO_2_/MOA) for which parameters such as the size of the quartz particles and the mixture ratio were optimized. The optimized stationary phase was used to effectively separate 9-methyladenine (9-MeA) from 1-methylthymine (1-MeT) and 1-methyluracile (1-MeU). Finally, theoretical calculations were performed to analyze the interaction energies of [Cu(UPrO)_2_(4,4′-bipy)_2_(H_2_O)] discrete entities with CO_2_ and with methylated nucleobases, to support both the selective disruption of the supramolecular interactions leading to the microstructural transformation above described and also the elution sequence of the methylated nucleobases in the chromatograms.

## 2. Materials and Methods

### 2.1. Reagents

Commercial reagents and solvents were procured from standard chemical suppliers: Uracil-1-propionic acid (UPrOH) 98%, Fluorochem (Glossop, UK) 4,4′-Dipyridyl (4,4′-bipy) 98%, Sigma-Aldrich (Darmstadt, Germany); CuSO_4_·5H_2_O > 98%, Prolabo (Camorino, Switzerland); sodium hydroxide 98.5% (NaOH) Scharlau (Barcelona, Spain); Glacial acetic acid (AcOH) 99.8% Carlo Erba Reagents (Val-de-Reuil, France); MeOH 99.8% and EtOH 99% Scharlau (Barcelona, Spain); and used as received. 

### 2.2. Characterization Techniques

Mineral oil was used to coat the selected crystals which were mounted on MiTeGen MicroMounts (from Ithaca, NY, USA). Single-crystal X-ray diffraction was carried out in a Bruker D8 KAPPA series II (from Karlsruhe, Germany) with APEX II area-detector system equipped with graphite monochromated Mo K_α_ radiation (λ = 0.71073 Å). The abundance of collected data enabled empirical absorption corrections (SADABS) [38] to be applied using multiple measurements of symmetry-equivalent reflections. SAINT program version 6.14 (Bruker AXS Inc.), [39] which applied corrections for Lorentz and polarization effects, was used to integrate raw intensity data frames. Space-group determination, structure solution, and refinement were carried out using the Bruker SHELXTL Software Package version 8.27B (Bruker AXS) [40]. By direct methods (SHELXS-97), [38] completed with different Fourier syntheses, the structures were solved and refined with full-matrix least-squares using SHELXS-97 minimizing ω(F_o_^2^ − F_c_^2^)^2^. Weighted R factors (R_w_) and all goodness of fit S are based on F^2^; conventional R factors (R) are based on F. All non-hydrogen atoms were refined with anisotropic displacement parameters. All scattering factors and anomalous dispersion factors are contained in the SHELXTL 6.10 program library. Table 1, Tables S1 and S2 contain the most relevant data acquisition and structure refinement information.

Infrared (FT-IR) spectra were recorded on a Perkin Elmer 100 spectrophotometer using a PIKE Technologies MIRacle Single Reflection Horizontal ATR Accessory (Bridgeport, CT, USA) from 4000 to 500 cm^−1^. 

Elemental analysis was performed on an elemental microanalyzer LECO CHNS-932 (Madrid, Spain). It works with controlled doses of O_2_ and a combustion temperature of 1000 °C.

Powder X-ray diffraction was carried out using a PANalytical X’Pert PRO (from Malvern, UK) Cu-Kα configuration (λ = 1.5418 Å) with θ/2θ geometry, equipped with a secondary monochromator and fast X’Celerator detector (Malvern, UK) which was used for general assays. Theoretical X-ray powder diffraction patterns were calculated using Mercury 2020.2.0 software by CCDC from the Crystallographic Cambridge Database (CSD). 

Thermogravimetric analysis (TGA) was made with a TGA Q500 Thermobalance provided with an EGA (Evolved Gas Analysis) furnace (TA instruments, Madrid, Spain) and a quadrupole mass spectrometer Thermostat Pfeiffer from Tecnovac (Madrid, Spain), to analyze gases produced from the sample. A Pt sample holder was used to deposit powder sample and N_2_ flow of 90 mL/min as purge gas, with a heating ramp from room temperature to 1000 °C at 10 °C/min.

Field-emission scanning electron microscopy (FESEM) images were taken on a JEOL JSM 7600F microscope (Croissy-sur-Seine, France). SEM–EDX images and EDX spectra were performed using a Hitachi S-3000N microscope with an ESED coupled to an INCAx-sight EDX analyzer (Krefeld, Germany). For the latter equipment, the samples were metalized with a 15 nm gold layer under a pressure of 10^−3^ Pa.

Magnetic measurements were performed in a Quantum Design MPMS-XL-5 SQUID magnetometer (San Diego, CA, USA) in the 2–300 K temperature range with an applied magnetic field of 0.1 T to the crystalline samples.

Adsorption measurements were completed on a Quantachrome Autosorb-iQ-MP (Florida, USA). Prior to N_2_ (Table 2) and CO_2_ adsorption measurements, all samples were outgassed under vacuum at 50 °C for 6 h. Nitrogen adsorption data were collected at 77 K, while carbon dioxide adsorption data were obtained at 273 and 293 K.

### 2.3. Theoretical Methodology and Computational Details 

All ab initio calculations were carried out by an adequate combination of the localized basis set Gaussian16 [41] and the plane-wave QUANTUM ESPRESSO atomistic simulation packages [42] to obtain optimized structures and characterize the interaction between the [Cu(UPrO)_2_(4,4′-bipy)_2_(H_2_O)] discrete entities, with CO_2_ and with different methylated nucleobases as well as to analyze the effect in these interactions of a water solvent reaction field [41]. In addition to this, to evaluate intermolecular interactions within the crystalline structure of the compound [42] by accounting periodic conditions.

### 2.4. Synthesis

#### 2.4.1. Synthesis of [Cu(UPrO)_2_(4,4′-bipy)_2_(H_2_O)] (CuUPrO) and [Cu(SO_4_)(4,4′-bipy)]_n_·2nH_2_O Single-Crystals

CuSO_4_·5H_2_O (0.1 g, 0.4 mmol), UPrOH (0.147 g, 0.8 mmol), NaOH (0.032 g, 0.8 mmol) and 4,4′-bipy (0.125 g, 0.8 mmol) were mixed in 12 mL of Milli-Q water (pH = 4.2) in a glass hydrothermal reactor and stirred for 10 min at room temperature. Then, the mixture was heated at 120 °C for 24 h, followed by a cooling ramp at ca. 0.083 °C/min. Following this, blue crystals altogether with a small amount of green crystals were obtained. The mixture was filtered off and manually separated, washed with water, ethanol, diethyl ether, and dried in air. Blue crystals were suitable for single-crystal X-ray diffraction studies (Figure S1 and Table 1, Tables S1 and S2), which belong to the compound CuUPrO (78% yield based on Cu). Chemical Analysis, calculated (found) % for C_34_H_31_CuN_8_O_9_: C, 53.79 (53.24); H, 4.12 (4.30); N, 14.76 (14.39). Characteristic IR bands (cm^−1^): 3262 (w), 1682 (s), 1591 (s), 1406 (s), 1298 (m), 1225 (m) and 809 (w). Green crystals belong to the published coordination polymer [Cu(SO_4_)(4,4′-bipy)]_n_·2nH_2_O [43] (yield 5% based on Cu). 

#### 2.4.2. Synthesis of [Cu(UPrO)_2_(4,4′-bipy)_2_(H_2_O)] (CuUPrO) in Bulk

A mixture of CuSO_4_·5H_2_O (0.1 g, 0.4 mmol), UPrOH (0.147 g, 0.8 mmol), NaOH (0.032 g, 0.8 mmol) and 4,4′- bipy (0.125 g, 0.8 mmol) was stirred in 19 mL of Milli-Q water (pH = 4.5) for 25 min at room temperature. The resulting blue suspension was filtered off, washed with water (15 mL × 3), ethanol (15 mL × 3), diethyl-ether (15 mL × 3), and dried in air (yield: 86% based on Cu). Chemical Analysis, calculated (found) % for C_34_H_31_CuN_8_O_9_: C, 53.79 (52.41); H, 4.12 (4.30); N, 14.76 (14.21); S, 0 (0.38). Characteristic IR bands (cm^−1^): 3262 (w), 1682 (s), 1591 (s), 1406 (s), 1298 (m), 1225 (m) and 809 (w) (Figure S2). PXRD pattern of powdery blue solid is depicted in Figures S3 and S4. 

#### 2.4.3. Synthesis of CuUPrO@MOG and CuUPrO@MOA

A total of 0.08 g of compound [Cu(UPrO)_2_(4,4′-bipy)_2_(H_2_O)] (CuUPrO) was scattered in 0.17 mL of glacial acetic acid and 4 mL of MeOH, followed by sonication in an ultrasound bath for 15 min and heated at 45 °C for another 15 min (37 kHZ, 380 W). Following this, a blue gel was obtained, CuUPrO@MOG. This gel was immersed in acetone for 48 h to allow the solvent exchange between methanol and acetone and to dilute the remaining acetic acid. Then, the gel was introduced into a filter paper box inside an autoclave to exchange acetone by liquid CO_2_ (five times, one hour per exchange). Finally, CuUPrO@MOA was obtained by raising the temperature of the reactor up to 40 °C (pressure increases accordingly up to ca. 80 bar) to obtain CO_2_ as a supercritical fluid. It was left at these conditions for half an hour, and later the pressure was driven down to atmospheric pressure by slowly releasing CO_2_ through the purge valve of the autoclave reactor. Chemical Analysis, calculated (found) % for theoretical (C_34_H_31_CuN_8_O_9_)·3CH_3_OH: C, 51.96 (52.04); H, 5.07 (4.84); N, 13.10 (13.33); S 0 (0.72). Characteristic IR (Figure S5) bands (cm^−1^): 3266 (w), 1683 (s), 1593 (s), 1404 (s), 1293 (m), 1227 (m) and 808 (w). PXRD of CuUPrO@MOA is depicted in Figure S6.

### 2.5. HPLC Column Design

Steel columns of 100 mm length and 4.6 mm diameter were filled with SiO_2_ microparticles and the CuUPrO@MOA. Different morphological SiO_2_ particles (manually grounded, grounded in a ball-miller, and commercial mesoporous microspheres) were employed using variable loads of CuUPrO@MOA (0, 2.5, 5, and 7.5 wt.%) which were used for column optimization (Table 3, Table 4 and Table 5). Prepared columns can work under moderate pressure (500–1500 psi) and broadly used HPLC conditions (hexane/isopropanol 90:10; flow 1.0 mL min^−1^).

## 3. Results and Discussion

### 3.1. Crystal Structure Description of CuUPrO

**Figure 1:**
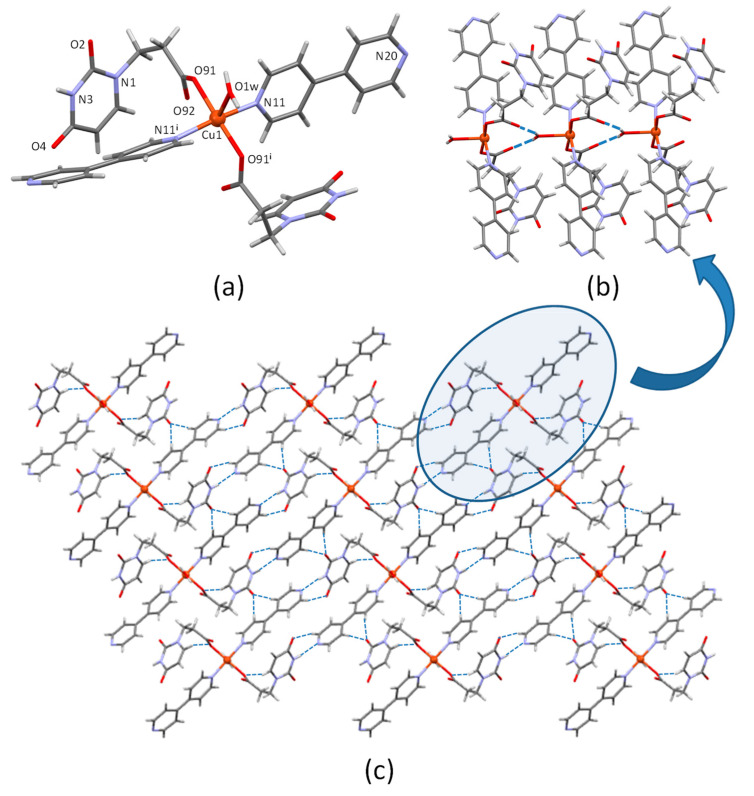
(**a**) Monomeric [Cu(UPrO)_2_(4,4′-bipy)_2_(H_2_O)] (CuUPrO) entity, (**b**) supramolecular chains through water-mediated hydrogen bonds (dashed lines), and (**c**) crystal packing of compound CuUPrO along the crystallographic b axis showing the hydrogen-bonding interactions involving the uracil residue.

Compound CuUPrO was obtained with a high yield in water both under room and hydrothermal conditions by the reaction of CuSO_4_·5H_2_O with UPrOH, NaOH, and 4,4′-bipyridine in a 1:2:2:2 stoichiometry. The NaOH was used to deprotonate the carboxylic group of the propionic acid. Suitable blue single-crystals for XRD structural characterization were obtained by hydrothermal reaction during 24 h at 120 °C. The resulting coordination compound crystallizes in the monoclinic C2/c space group (Figure 1 and Figure S1, Table S1). It consists of discrete [Cu(UPrO)_2_(4,4′-bipy)_2_(H_2_O)] entities. The Jahn–Teller effect of the Cu(II) center is responsible for its elongated square-pyramidal coordination-geometry (Figure 1a). The bond distances in the basal plane lie around 1.96 and 2.03 Å involving two oxygen atoms from two syn-coordinated uracil-1-propionate ligands and two nitrogen atoms from two 4,4′-bipy. The apical position with a significantly longer bond distance (2.21 Å) is occupied by one oxygen atom from a water molecule. Elected coordination bond lengths and angles are gathered in Table S2.

The water molecule establishes a symmetry-related double hydrogen-bond (Ow-H···O92) with the uncoordinated carboxylate oxygen atoms (Table 1) from an adjacent entity (“head-to-tail” interaction) to provide a 1D spreading supramolecular chain. These chains are further connected among them through hydrogen bonds involving the uracil residues, which establishes a complementary hydrogen bond interaction with the uncoordinated edge of the 4,4′-bipy ligand (C19_bipy_-H···O4_uracil_ and N3_uracil_-H···N20_bipy_) groups, “side-to-side” interactions. The uracil O2 position also receives two hydrogen bonds from the 4,4′-bipy ligand aromatic C-H groups (C15_bipy_-H···O2_uracil_ and C22_bipy_-H···O2_uracil_). There is no evidence of any close contact between the aromatic ring of the organic ligands that could suggest any kind of π-stacking interaction, probably because the abundance of hydrogen-bond donor and acceptor positions available in this compound precludes the necessity of any further stabilization through this kind of usually less robust supramolecular interactions. 

**Figure 2:**
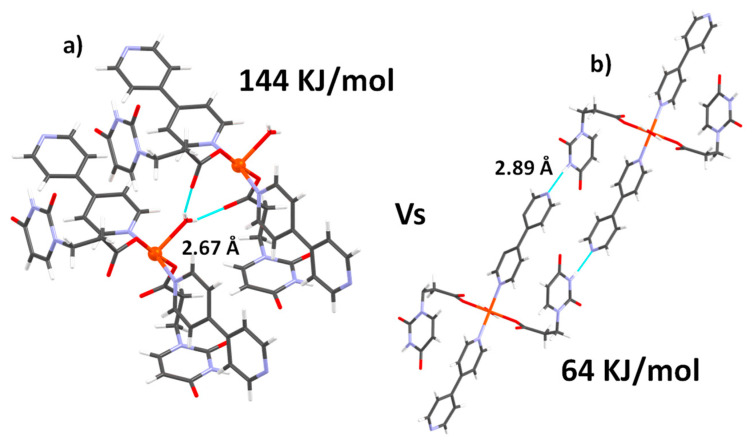
Pictorial sketch of the two non-equivalent stabilizing cohesive interactions separately within the [Cu(UPrO)_2_(4,4′-bipy)_2_(H_2_O)] compound: (**a**) “head-to-tail” interaction and (**b**) “side-to-side” interaction between discrete entities. Interaction energies and representative bond lengths are also indicated.

DFT calculations on the interaction energies between the [Cu(UPrO)_2_(4,4′-bipy)_2_(H_2_O)] discrete entities reveal the highly anisotropic nature within the supramolecular interaction network, being the hydrogen bonding interaction involving the coordinated water molecule far stronger than the lateral supramolecular interactions. Figure 2 shows the result of these calculations, by which we have evaluated, under periodic conditions, the two major stabilizing cohesive interactions within the crystal. On one side, the “head-to-tail” interaction stabilizing the mono-dimensional chains (Figure 2a) results in a cohesive energy of 144 KJ/mol per discrete entity, involving a double strong hydrogen bond interaction (bond-length: 2.67 Å), whilst the “side-to-side” interaction (Figure 2b) results in an interaction energy of 64 KJ/mol per discrete entity for two less strong symmetry-related N-H···N hydrogen bonds (bond-length: 2.89 Å). These values manifest, as mentioned, a high anisotropy between the cohesive stabilization forces, which would lead to a prevalence of the mono-dimensional disposition of the subunits by means of “head-to-tail” interactions overriding the “side-to-side” cohesion. This fact agrees with the below described formation of nanofibers which may have its origin in a plausible electrostatic “lateral” piling of the prevailing mono-dimensional chains.

**Figure 3:**
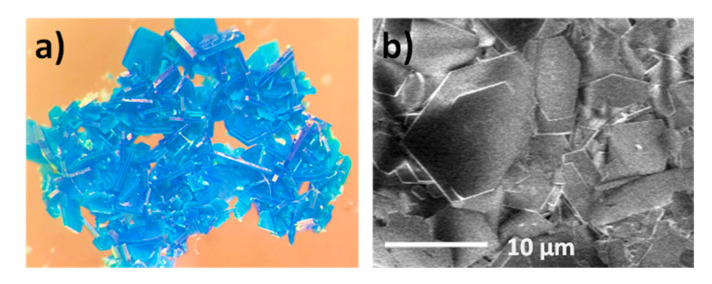
Optical microscope image of CuUPrO single-crystals (**a**). SEM images of powdery CuUPrO (**b**).

Scanning electron microscopy (SEM) images of compound CuUPrO indicate a plate-like crystal morphology with several microns width and 50 ± 30 nm height (Figure 3). It should be noted the presence of trace levels of [Cu(SO_4_)(4,4′-bipy)]_n_·2nH_2_O [43] during room-temperature synthesis of CuUPrO, was confirmed by a detailed study through XRD and SEM–EDX. The complete characterization of CuUPrO is depicted in Figures S7–S16 (see Supplementary Materials).

### 3.2. Preparation of MOG and MOA 

**Scheme 1:**
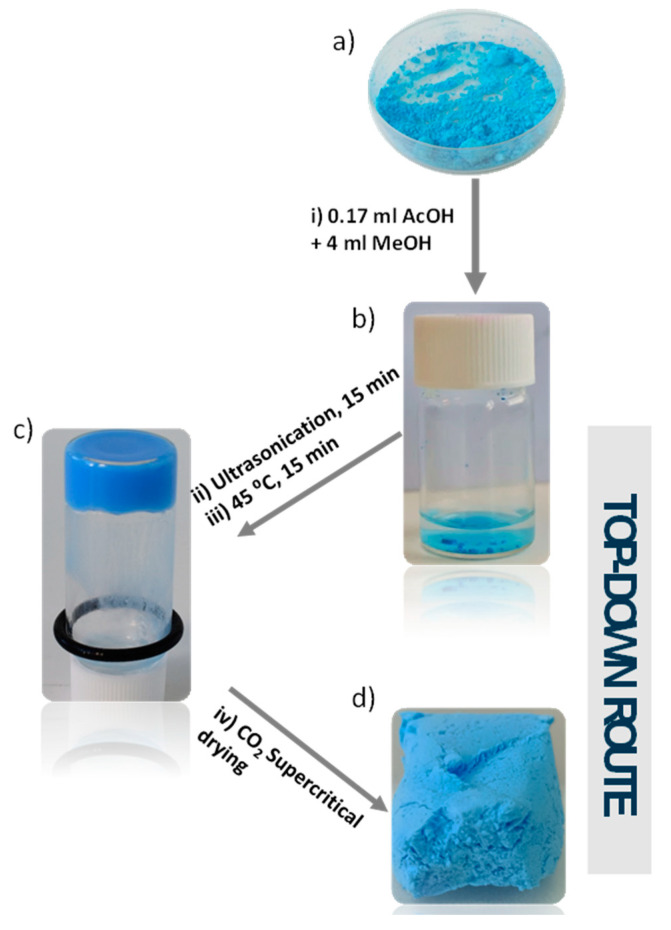
Illustration scheme of MOG and MOA process in which powdery CuUPrO (**a**) is dispersed in a mixture of acetic acid (AcOH) and methanol (MeOH) (**b**), transformed into CuUPrO@MOG after applying ultrasound sonication for 15 min and heating at 45 °C (**c**), and finally, transformed into CuUPrO@MOA by its CO_2_ supercritical drying (**d**).

In general, the gelation process induced via top-down approaches has been rarely studied [44], and focusing on metal–organic gels (MOGs), as far as we know, this work represents the first example of this kind of approach. The optimized procedure (Tables S4 and S5) implies sonicating 80 mg of CuUPrO in 4 mL of methanol containing 0.17 mL of acetic acid for 15 min and subsequent heating of the mixture at 45 °C (Scheme 1) for another 15 min. Unlike most of the reported works [45], the plate-like morphology of the particles comprising the CuUPrO@MOG (Figure S11c) was drastically transformed into very long (lengths above the micrometer) and relatively homogeneous nanofibers with 24 ± 6 nm diameter (Figures S11d and S12) during the CO_2_ supercritical drying procedure, which are entangled together to provide CuUPrO@MOA (Scheme 1d).

**Figure 4:**
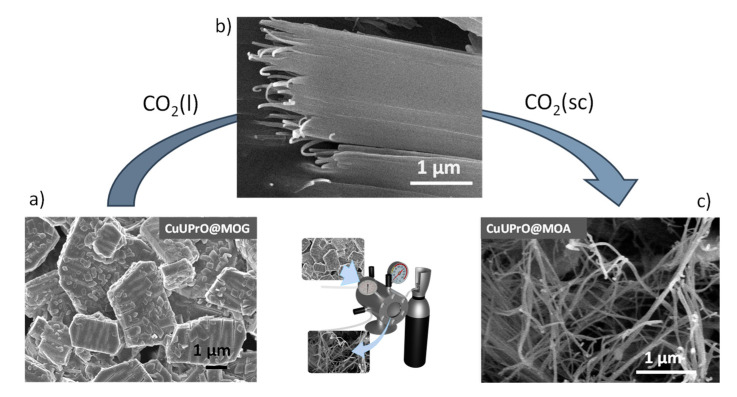
FESEM images of the transformation process from hexagonal platelets of CuUPrO (**a**) after applying the top-down approach, in which an unravelling process of crystals is observed (**b**) when they are subjected to liquid CO_2_ for 5 h at 50 bar at 10 °C. Finally, the transformation ends when CO_2_ transforms from liquid to supercritical state (40 °C, 80 bar), resulting in curled nanofibers (**c**).

Surprisingly, CuUPrO@MOA retains the CuUPrO structure with almost no loss of crystallinity (Figure S6) and several experiments were performed to obtain a deeper insight into the mechanism leading to this microstructural transformation (Figure S13). The first clear evidence comes from the liquid CO_2_ performed over pristine bulk samples not subjected to the gelation procedure, which did not exert any change on the hexagonal platelets (Figure S14). In contrast, the same platelets in the gelled sample produced the above-mentioned transformation. Consequently, it can be inferred that the presence of small amounts of acetic acid (acetic acid solely as the solvent produces entire dissolution of CuUPrO) remaining from the gelation procedure, despite the gel being previously subjected to exchange solvent with acetone for 48 h, plays a crucial role in the transformation mechanism as well as the starting copper salt used since no gel is formed when it is employed with a starting salt different from CuSO_4_ (Figures S4 and S10 and Table S3). Additional information arose from the bulk CuUPrO and acetone-soaked CuUPrO@MOG gel samples subjected to 50 bar in an argon atmosphere (Figure S13b,d). The bulk sample does not show any substantial change in argon or liquid CO_2,_ but the CuUPrO@MOG samples display clear evidence of an incipient unravelling process (Figure 4b) of the initial platelet-shaped crystals (more evident for liquid CO_2_ than for argon) (Figure S13e,d). However, the unravelling is only completed under CO_2_ supercritical conditions in which the initial platelets have completely disappeared, and the resulting fibers (Figure 4c) are curled into a mesoporous and light aerogel.

The reasons for this behavior could be identified in the acetic acid molecules remaining from the gelation procedure, which play an initiator/catalyst role due to its capacity to reversibly protonate the heteroatoms of both the uracil and pyridine residues of the ligands, leading to a disruption of the less strong lateral supramolecular interactions. The latter would start separating supramolecular chains, observable as parallel cracks in the crystal surface. However, to allow progression of this process, the solvent must interact strongly enough with the lateral residues of the supramolecular chain to keep the emerging fibers separated. At this late stage of the mechanism, the solvation properties of the solvent are crucial since poor solvating solvents do not allow the initial crackings to progress (acetone pressurized at 50 bar under an argon atmosphere). In the case of more interactive conditions (liquid CO_2_) the fibers begin emerging from the platelets to finally complete disaggregation of the nanofibers under better solvating conditions provided by supercritical CO_2_, which takes place without any retention of the initial platelets. Supercritical CO_2_ provides the perfect balance of the required solvation capabilities to disrupt only the lateral weaker supramolecular interactions leaving the stronger water-mediated hydrogen bonding synthons unaltered that hold together the complexes within the 1D supramolecular chain. The use of more solvating capable solvents, such as water, gives rise to a complete disruption of all supramolecular interactions and to the sample solubilization. Theoretical calculations on the interaction energies between the uracil and pyridine residues of the ligands with CO_2_ provide values (Figure S17) close to or greater than those of the lateral interchain interactions but still weaker than the synthon merging the supramolecular chain. 

Several previous studies confirm the high degree of morphological tailorability of coordination compounds and other soft materials depending on the reaction time, solvent, light induction, pressure, ultrasound-induction, and temperature, among other parameters [19,46]. However, as far as we know, no studies have reported such microstructural changes during the CO_2_ supercritical drying procedure [47,48].

### 3.3. Analysis of CuUPrO@MOA Adsorption Properties

The N_2_ (77 K) adsorption curve resembles a type II isotherm (Figure S15a), indicating a dominating macroporosity. Data (specific surface area, pore volume, and lamella thickness values) obtained from the adsorption isotherm are shown in Table 2. CuUPrO@MOA, triples the surface area values of the recently reported CuUAcO@MOA (67 vs. 21 m^2^ g^−1^) [22]. As CuUPrO lacks intrinsic porosity, a higher surface area value can be ascribed to smaller particle size of the nanofibers (section: 12 × 23 nm) with respect to the platelets (thickness: 50 nm, lateral dimensions of several microns). 

Moreover, CuUPrO@MOA exhibits the thinnest particle and certain contribution of microporosity which can be ascribed to the presence of narrow cavities among the nanofibers in the aerogel microstructure. 

In this respect, quenched solid density functional theory (QSDFT; slit-pores) was used for modeling pore size distribution (PSD) as implemented in ASiQwin software (V5.2, 2017, Quantachrome, Boynton Beach, FL, USA). The low R^2^-factors (0.1%) and the good agreement between DFT (69.1 m^2^ g^−1^) and BET area (67.1 m^2^ g^−1^) values support the suitability of the selected DFT kernel. According to the trend inferred from the above-mentioned absolute pore volume values (V_micro_ and V_T_) and the PSD plots (Figure S15b) it was confirmed that CuUPrO@MOA exhibits a great microporous contribution (pores < 2 nm).

In the second instance, the characterization of the porosity was completed with the measurements of CO_2_ adsorption isotherms at 273 and 293 K to estimate the isosteric heats of adsorption (Q_st_) (Figure S16). The Q_st_ value was calculated using the modified Clausius–Clapeyron equation [49], which is the limit of zero loading approach to 39.6 KJ mol^−1^. This value is comparable to those reported for well-known MOFs MIL-101(Cr) (44 KJ mol^−1^), MOF-74(Ni) (42 KJ mol^−1^), bioMOF-1(Zn) (35 KJ mol^−1^), MIL-53(Al) (35 KJ mol^−1^), and HKUST-1(Cu) (35 KJ mol^−1^) [50]. The isosteric heat values of the herein CuUPrO@MOA can be regarded as relatively high values when compared with inorganic aerogels and metal–organic aerogels, [51] which can be probably related to amino functionality of the coordination polymer arising from the nucleobase residue and the microstructural microporosity contribution.

### 3.4. CuUPrO@MOA as Stationary Phase for HPLC Column Design

To create a composite filler capable of adequately performing in HPLC conditions, a total of six different columns (A–E, Table 3) were prepared in which several parameters were analyzed: ball-miller or manually ground SiO_2_ particles as cofiller, CuUPrO, or CuUPrO@MOA, greater or lower CuUPrO@MOA content. The column performance was analyzed using two aromatic representative molecules (toluene and binol) for separations of various aromatic compounds such as toluene and binol, which was evaluated. All latter prepared columns (except column B and column E) provide effective baseline separation of these representative molecules (toluene and binol), while retention is inexistent using only SiO_2_ particles as filler, indicating the efficiency of our aerogel under these conditions, as stationary phase.

The results of columns A and B indicate that for the same CuUPrO@MOA amount columns filled with ball-miller ground SiO_2_ (particle size < 5 µm) show better performance than the column filled with manually ground SiO_2_ (particle size: 10–30 µm) as shown in Figure S18. Regarding the CuUPrO@MOA percentage in the HPLC column, the best resolution parameters are obtained for column C (5% of CuUPrO@MOA). This result is promising as it indicates that a functional HPLC column stationary phase can be achieved simply using a modest amount of the active material. Moreover, it is worth noting the effect of the material microstructure on column performance when columns C (filled with CuUPrO@MOA) and E (filled with the bulk CuUPrO) are compared. The greater surface area provided by the nanofibers of the aerogel clearly boosts the performance of the HPLC column. 

Thereafter, the capability of the CuUPrO@MOA filled HPLC columns to separate compounds with similar polarities (binol and 2-naphthol) was tested (Table 4). Although in none of these columns complete line base separation was achieved, the separation factor (α) and resolution (R_s_) values indicate a better performance of column C with 5.0% of CuUPrO@MOA (Table 4: α = 6.00 and R_s_ = 1.00). The size-based and interaction-based separation implies higher retention for binol than the less bulky and less hydrogen bonding capable 2-naphthol.

**Figure 5:**
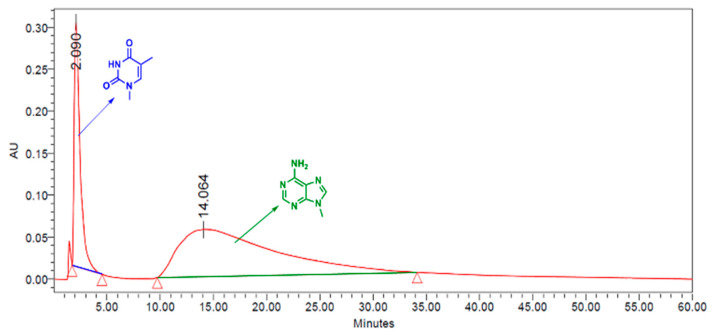
9-MeA and 1-MeT separation achieved with the quartz/metal–organic gel composite HPLC column C.

Column C, which exhibited the best separation performance, was used to determine the retention time for a series of methylated nucleobases. The results indicate that N9-methyladenine (9-MeA) (t_R_ = 14.064 min) could be efficiently separated from N1-methylthymine (1-MeT) (t_R_ = 2.151 min) and N1-methyluracil (1-MeU) (t_R_ = 3.130 min). To confirm this, a mixture of 9-MeA and 1-MeT was injected in the column, using a more polar hexane/isopropanol 80:20 ratio (flow 1.0 mL min^−1^) to reduce the peak tailing, giving rise to the chromatogram depicted in Figure 5. The obtained separation (α = 21.29) and resolution (R_S_ = 1.28) indicate a good performance on this task.

In order to analyze the interaction energy of the CuUPrO entities and the selectivity in the stabilization of the different 9-MeA, 1-MeT, and 1-MeU nucleobases, a battery of DFT-based calculations were carried out. Two favorable adsorption sites per CuUPrO entity involving different sides of the uracil residue (configuration I: C2=O2 and N3-H side; configuration II: N3-H and C4=O4) were detected (see further details on all the different configurations tested in Suppl. Info., Figures S19–S22). The obtained hydrogen bond distances for the adsorbed species ranged between 2.75 and 2.95 Å, typical of the Watson–Crick nucleobase-pair interactions. Table 5 summarizes the main findings obtained from these calculations. Interestingly, 9-MeA nucleobase yields the strongest interaction energy per molecule, with the values of 59.7 KJ/mol and 42.4 KJ/mol for configurations I and II, respectively. Similar configuration I and II arrangements for 1-MeT and 1-MeU molecules provide adsorption energies that are roughly half those of 9-MeA. Performing the same calculations with a water solvent reaction field modifies the energy values less than 5% and does not alter the energetic ordering.

The chromatogram retention times seem to follow the expected order of elution based on these theoretical calculations: 1-MeT < 1-MeU << 9-MeA, which agrees with the expected preference towards canonical A–U base-pairing interaction.

## 4. Conclusions

A novel top-down approach was provided based on the selective disruption through CO_2_ supercritical drying of the anisotropic supramolecular interactions to transform a conventional microcrystalline compound into a far more porous nanofibrillar material. The anisotropy of the non-covalent interactions in [Cu(UPrO)_2_(4,4′-bipy)_2_(H_2_O)] complex with extremely strong hydrogen-bonds in one direction and relatively weak lateral interactions provides an opportunity for their selective disruption based on the interaction capabilities of the solvent (supercritical CO_2_) and the presence of small amounts of acid species (acetic acid) as initiators. Based on this approach, a profound microstructural change can be achieved by dismantling nanometric wide strips from the original micrometric platelet-shaped crystals. This microstructural transformation boosts the inherent selective recognition capabilities of the uracil residue within the [Cu(UPrO)_2_(4,4′-bipy)_2_(H_2_O)] units providing an increased surface for the interaction with the analyte. Such features can prove to be extremely useful in applications such as HPLC chromatography, according to the successful results obtained in the separation of methylated nucleobases.

## Figures and Tables

**Table 1:** Hydrogen bonding interactions (Å, °) for CuUPrO ^[a]^.

D-H···A ^[b]^	H···A	D···A	D-H···A
N3-H···N20i	2.05	2.900(4)	172.5
O1w-H···O92ii	1.91	2.677(3)	156.0
C6-H···O91iii	2.42	3.279(4)	153.0
C15-H···O2iv	2.38	3.302(4)	170.1
C22-H···O2iv	2.27	3.200(5)	174.4
C19-H···O4v	2.41	3.333(5)	169.4

^a^ Symmetry codes: (i) −x + 3/2, −y + 1/2, −z + 1; (ii) x, y − 1, z; (iii) x, y + 1, z; (iv) x, −y, z + 1/2; (v) −x + 3/2, −y + 3/2, −z + 1. ^b^ D: donor; A: acceptor.

**Table 2:** Surface area and pore volume data.

Sample	S_BET_ ^a^ (m^2^ g^−1^)	S_micro_ ^b^ (m^2^ g^−1^)	V_micro_ ^b^ (cm^3^ g^−1^)	V_T_ ^c^ (cm^3^ g^−1^)
CuUPrO@MOA	67.1	47.1	0.019	0.065

^a^: BET specific surface area. ^b^: micropore surface area (S_micro_) and volume (V_micro_) are estimated from the t-plot calculation. ^c^: total specific pore volume (VT) is computed at P/P_0_ = 0.99 to account for pores smaller than 200 nm, respectively.

**Table 3:** Column compositions and resolution values for toluene/binol separation ^a^.

Composition			t_R_ (min)		w_h/2_ (s)		R_s_
	SiO_2_ Grinding	MOA (%)	Toluene	Binol	Toluene	Binol	
A	manually	5.0	1.525	2.690	15	37	1.59
B	ball-miller	2.5	1.516	2.627	23	43	1.19
C	ball-miller	5.0	1.414	3.185	15	50	1.93
D	ball-miller	7.5	1.598	4.180	36	98	1.36
E ^b^	ball-miller	5.0	1.597	2.022	10	11	1.43

^a^ Resolution R_s_ = 1.18*(t_R2_ − t_R1_)/(w_h/2,1_ + w_h/2,2_) where t_R_ is the retention time measured at maximum intensity; w_h/2_ is the peak width at the half-height. ^b^ CuUPrO is used instead of CuUPrO@MOA.

**Table 4:** Column compositions and values of resolution (R_s_) for 2-naphthol and binol separation.

Composition			t_R_ (min)		w_h/2_ (s)		α ^a^	R_s_ ^b^
	SiO_2_ Grinding	MOA (%)	2-Naphthol	Binol	2-Naphthol	Binol		
B	ball-miller	2.5	1.696	2.433	49	27	5.09	0.60
C	ball-miller	5.0	1.686	3.045	70	28	6.00	1.00
D	ball-miller	7.5	2.045	3.843	81	47	5.02	0.95

^a^ Separation factor α = (t_R2_ − t_0_)/(t_R1_ − t_0_) with t_0_ representing the retention time for toluene which it can be assumed is not retained. ^b^ Resolution R_s_ = 1.18(t_R2_ − t_R1_)/(w_h/2,1_ + w_h/2,2_) where t_R_ is the retention time measured at maximum intensity; w_h/2_ is the peak width at the half-height.

**Table 5:** Interaction energy per molecule (in KJ/mol), E_int_/molecule, between CuUPrO and methylated nucleobases for one molecule per unit cell and for two different adsorption configurations.

	Configuration I (E_int_/molecule, KJ/mol)	Configuration II (E_int_/molecule, KJ/mol)
9-MeA	59.7	42.4
1-MeT	30.8	20.2
1-MeU	31.8	22.16

Positive sign of E_int_ represents favorable bonding.

## Data Availability

Data presented in this article is available on request from the corresponding author.

## Supplementary Materials

The following are available online at https://www.mdpi.com/article/10.3390/nano12040675/s1, Figure S1: Asymmetric unit of compound CuUPrO showing the thermal ellipsoids at 50% probability level, Figure S2: IR spectra of [Cu(UPrO)_2_(4,4′-bipy)_2_(H_2_O)] (CuUPrO), Figure S3: PXRD patterns of [Cu(UPrO)_2_(4,4′-bipy)_2_(H_2_O)] (CuUPrO). Blue line corresponds to the experimental data and black line corresponds to simulated data, Figure S4: PXRD patterns of [Cu(UPrO)_2_(4,4′-bipy)_2_(H_2_O)] (CuUPrO), from CuSO_4_·5H_2_O as starting salt, and [Cu(SO_4_)(4,4′-bipy)]_n_·2nH_2_O. Blue and black lines correspond to the experimental and simulated data, respectively. Green line corresponds to the simulated data of [Cu(SO_4_)(4,4′-bipy)]_n_·2nH_2_O. Signed peaks (9.1, 9.9, 12.2, 16.3 and 24.7°) indicate the presence of impurities of [Cu(SO_4_)(4,4′-bipy)]_n_·2nH_2_O in CuUPrO blue solid, Figure S5: IR spectra of CuUPrO@MOA (black line) and compound CuUPrO (blue line), Figure S6: PXRD patterns of CuUPrO@MOA (black line) and compound CuUPrO (blue line), Figure S7: Thermal variation of χ_m_T (left) and M (right) for compounds CuUPrO (a) and CuUPrO (b), Figure S8: Thermal stability of compound CuUPrO in bulk, synthesized from CuSO_4_ Thermogram signals: black and red line (weight (TG) and derivate weight (DTG), respectively) represent the stages of the losses produced. Multicolor-labelled lines represent the ion current associated with each mass lost in each stage, Figure S9: Thermal stability of compound CuUPrO@MOA. Thermogram signals: black and red line (weight (TG) and derivate weight (DTG), respectively) represent the stages of the losses produced. Multicolor-labelled lines represent the ion current associated with each mass lost in each stage, Figure S10: SEM-EDX images (a–c) for compound CuUPrO in bulk, synthesized from CuSO_4_·5H_2_O, Figure S11: FESEM images of polycrystalline compound CuUPrO (a) and its transformation during sonication process (b), after 45 °C and its exchange with acetone (c) and finally, after its drying with supercritical CO_2_ (d), Figure S12: Statistical dimension based on the diameter of CuUPrO@MOA nanofibers, Figure S13: Schematic illustration of the experiments performed on pristine CuUPrO (a); after being subjected to Ar(g) for 5h at 50 °C (b) and after applying the gel method and exchanging with acetone to obtain CuPrO@MOG (c). Upon this treatment, CuPrO@MOG is going through Ar(g) for 5 h at 50 °C (d), liquid CO_2_ for 5 h at 50 bar and 10 °C (e) and finally, when liquid CO_2_ goes to supercritical state (40 °C, 80 bar) (f), Figure S14: FESEM images of CuUPrO dispersed in methanol and subjected to liquid CO_2_ for 5 h at 50 bar and 10 °C (a) and under Argon for 5 h at 50 bar at room temperature (b), Figure S15: N_2_ adsorption isotherms (77 K) (a) and pore size distribution histogram of CuUPrO@MOA (b), Figure S16: CO_2_ adsorption isotherm of CuUPrO@MOA at 273 and 293 K (a) and isosteric heats vs CO_2_ loading (b), Figure S17: Pictorial sketch of the three most reactive sites upon the adsorption of CO_2_ on a CuUPrO unit labelled as A, B and C, Figure S18: SEM images of the sample polydisperse in sizes. Grinding (especially the ball mill) reduces the maximum particle size, >40 µm for neat sand (A), 10–30 µm for hand milled sand (B), <5 µm for ball milled sand (C) and increases the fraction of smaller particles (<0.5 µm) and reduces qualitatively the polydispersion, Figure S19: Pictorial sketch of a DFT-optimized two-entities model of CuUPrO to interact with the three methylated nucleobases 9-MeA, 1-MeT and 1-MeU (gas-phase structure), Figure S20: Different DFT-optimized configurations of 9-MeA adsorbed on the CuUPrO discrete entities with two different adsorption configurations. Two units are shown for a better appreciation of the longitudinal “head-to-tail” monodimensional arrangement. Interaction energy per MeA is also indicated in each case, Figure S21: Different DFT-optimized configurations of 1-MeT adsorbed on the CuUPrO discrete entities with two different adsorption configurations. Two units are shown for a better appreciation of the longitudinal “head-to-tail” monodimensional arrangement. Interaction energy per 1-MeT is also indicated in each case, Figure S22: Different DFT-optimized configurations of 1-MeU adsorbed on the CuUPrO discrete entities with two different adsorption configurations. Two units are shown for a better appreciation of the longitudinal “head-to-tail” monodimensional arrangement. Interaction energy per 1-MeU is also indicated in each case, Table S1: Asymmetric unit of compound CuUPrO showing the thermal ellipsoids at 50% probability level, Table S2: Selected bond lenghts (Å) and angles (°) for compound CuUPrO, Table S3: Energy-dispersive X-ray analysis for compound CuUPrO in which a corresponds to analysis in Figure S10a,b corresponds to FigureS10 b and c corresponds to Figure S10c. All results are in atomic %, Table S4: Conditions of gel formation for 0.08 g of compound CuUPrO and 4 ml of solvent. UG: unconsolidated Gel; G gel; C: colloidal suspension; P: precipitate; S: solution, Table S5: Stability of CuUPrO@MOG versus different organic solvents, temperature and acetic acid amount. UG: unconsolidated Gel; G: gel; C: colloidal suspension; P: precipitate; S: solution. Refs. [52,53,54,55,56,57,58,59,60] are cited in the supplementary materials.
