# Supplementary material for: Innovative Microstructural Transformation upon CO2 Supercritical Conditions on Metal-Nucleobase Aerogel and Its Use as Effective Filler for HPLC Biomolecules Separation
Source: Nanomaterials (Basel). 2022 Feb 17;12(4):675. doi: 10.3390/nano12040675 (PMC8880480; doi:10.3390/nano12040675)
Supplement: Supplementary file 1 [file nanomaterials-12-00675-s001.zip › nanomaterials-1576953-supplementary.pdf]

nanomaterials

Supplementary Materials

S1. Crystal Structure Data for CuUPrO

S2. Characterization Data

S3. Morphological Study and EDX Analysis of CuUPrO and CuUPrO@MOA

S4. Gel Formation Conditions

S5. Adsorption Study

S6. Theoretical Methodology and Computational Details

S7. Separation of Nucleobases by HPLC: Stationary Phase Column and DFT Calculations

S1. Crystal Structure Data for CuUPrO

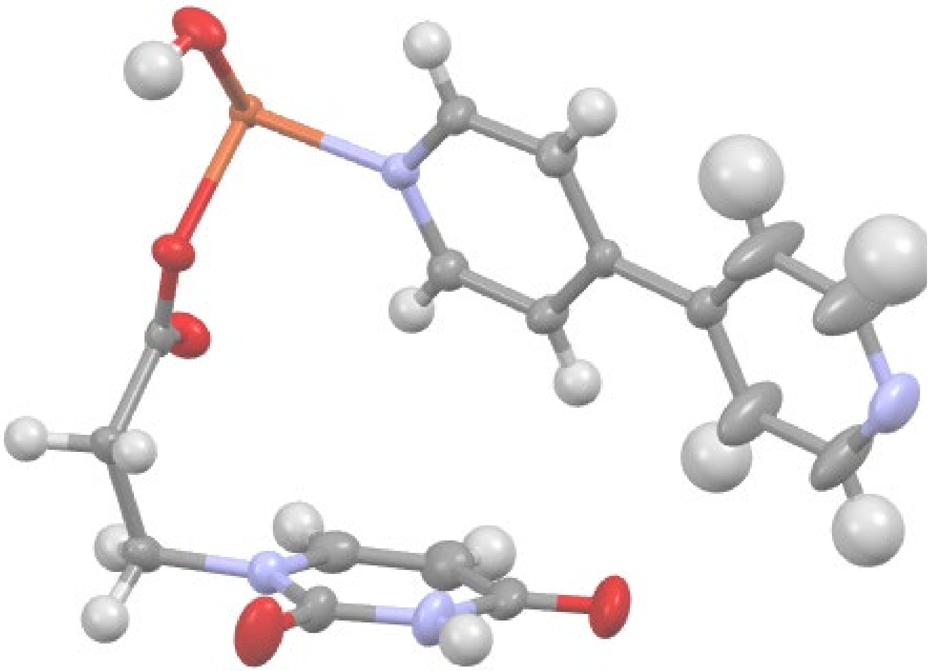

Figure S1. Asymmetric unit of compound CuUPrO showing the thermal ellipsoids at 50% probability level.

Table S1. Single-Crystal Data and Structure Refinement Details.

	CuUPrO
Formula	$C_{34}H_{31}CuN_8O_9$
w (g mol^{-1})	759.21
shape	plate
dimensions (mm)	0.19x0.12x0.08
T (K)	296(2)
space group	*C2/c*
a (Å)	36.9323(18)
b (Å)	5.9120(3)
c (Å)	16.9783(8)
β (º)	115.694(3)
V (Å^3)	3340.6(3)
Z	4
μ (mm^{-1})	0.723
D_{calc} (g cm^{-3})	1.510
independent reflns	3057
reflns [I> 2σ(I)]	2319
R_{int}	0.0773
Parameters	237
$\Delta\varrho_{max,min}$ (e Å^{-3})	0.534, –0.307
R indexes [a] [I> 2σ(I)]	R1 = 0.0419
	wR2 = 0.0843
R indexes (all data)	R1 = 0.0657
	wR2 = 0.0937

[a] $R1 = \Sigma||F_o| - |F_c||/\Sigma|F_o|$; $wR2 = [\Sigma[w(F_o^2 - F_c^2)^2]/[\Sigma(F_o^2)^2]]^{1/2}$ where $w = 1/[\sigma^2(F_o^2) + (AP)^2 + (BP)]$ with A = 0.0355 and B = 4.9975.

Table S2. Selected bond lenghts (Å) and angles (°) for compound CuUPrO.

Cu1-N11	2.032(2)	N11-Cu1-N11^{i}	169.18(13)
Cu1-O91	1.9604(17)	N11-Cu1-O91	89.48(8)
Cu1-O1w	2.212(3)	N11-Cu1-O91^{i}	90.85(8)
		N11-Cu1-O1w	95.41(7)
		O91-Cu1-O91^{i}	176.47(12)
		O91-Cu1-O1w	88.23(6)

Symmetry code: i) −x + 1, y, −z + 1/2.

S2. Characterization Data

S2.1. XRD Patterns and ATR-IR Spectra

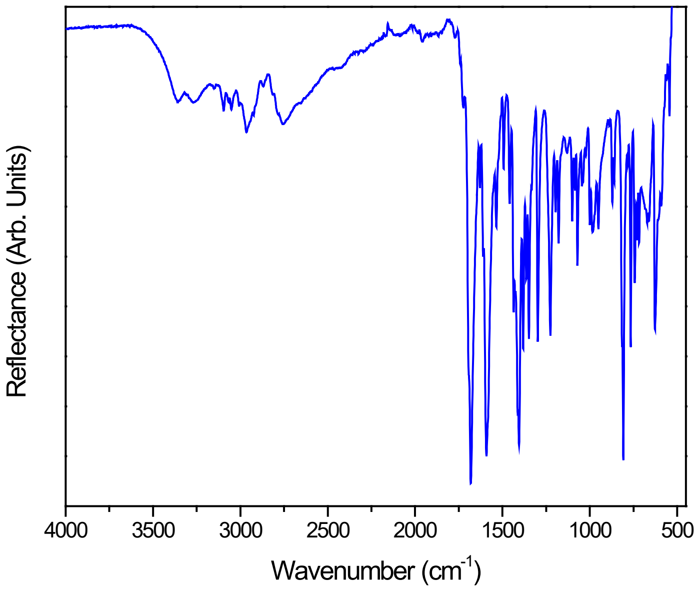

Figure S2. IR spectra of [Cu(UPrO)$_2$(4,4′-bipy)$_2$(H$_2$O)] (CuUPrO).

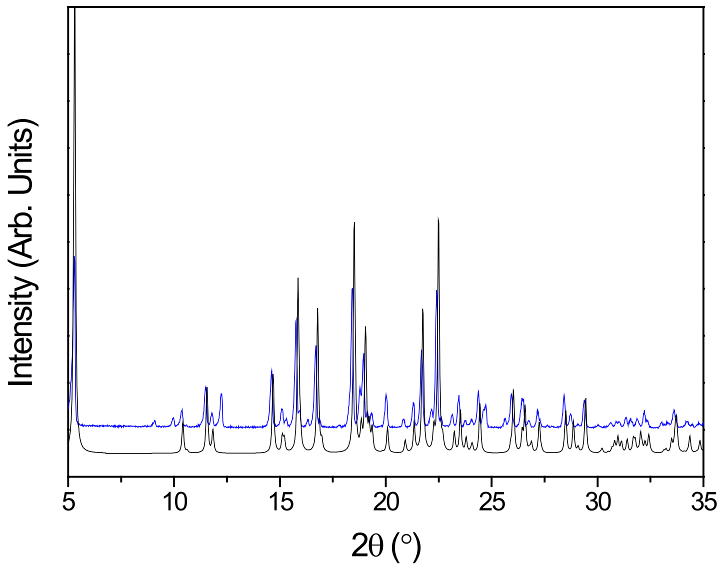

Figure S3. PXRD patterns of [Cu(UPrO)$_2$(4,4′-bipy)$_2$(H$_2$O)] (CuUPrO). Blue line corresponds to the experimental data and black line corresponds to simulated data.

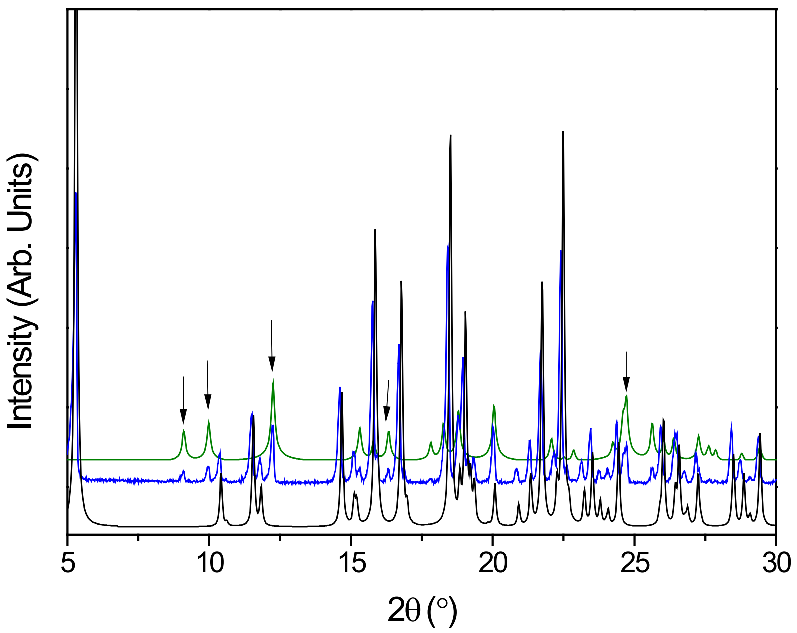

Figure S4. PXRD patterns of [Cu(UPrO)$_2$(4,4′-bipy)$_2$(H$_2$O)] (CuUPrO), from CuSO$_4$·5H$_2$O as starting salt, and [Cu(SO$_4$)(4,4′-bipy)]$_n$·2nH$_2$O [43]. Blue and black lines correspond to the experimental and simulated data, respectively. Green line corresponds to the simulated data of [Cu(SO$_4$)(4,4′-bipy)]$_n$ ·2nH$_2$O. Signed peaks (9.1, 9.9, 12.2, 16.3 and 24.7°) indicate the presence of impurities of [Cu(SO$_4$)(4,4′-bipy)]$_n$·2nH$_2$O in CuUPrO blue solid.

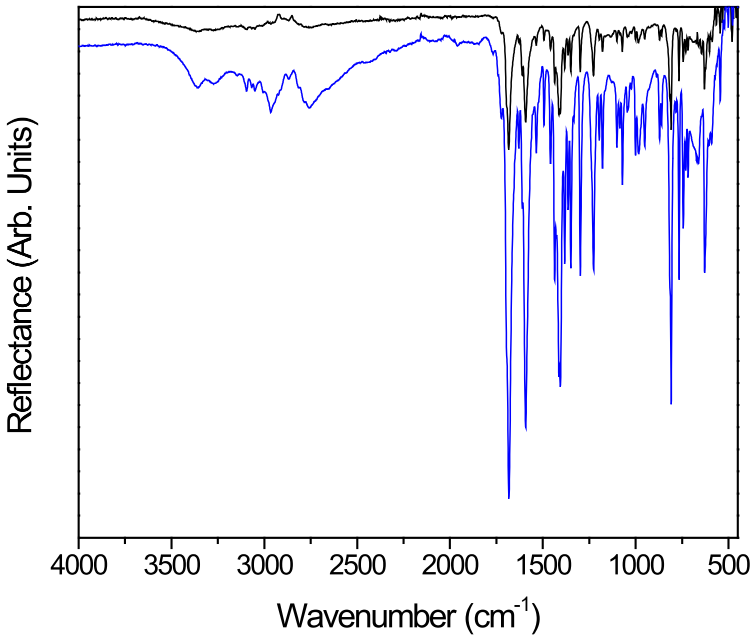

Figure S5. IR spectra of CuUPrO@MOA (black line) and compound CuUPrO (blue line).

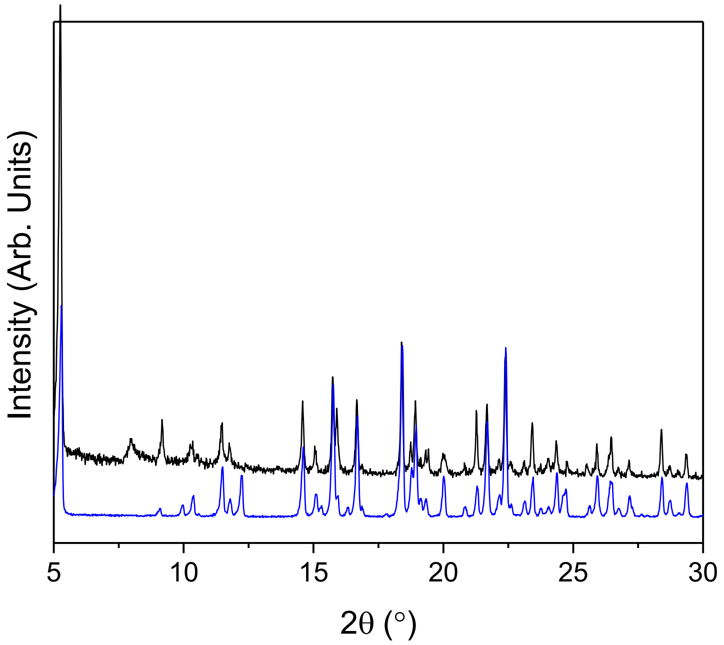

Figure S6. PXRD patterns of CuUPrO@MOA (black line) and compound CuUPrO (blue line).

S2.2. CuUPrO, CuUPrO@MOA Magnetic Properties

The product of the molar magnetic susceptibility per Cu(II) and temperature ($\chi_m T$) for compounds CuUPrO and CuUPrO @MOA show, at 300 K, values of ca. 0.43 and 0.49 cm^3 K mol^{-1} respectively (Figure S8). These values are clearly close to the expected value for an isolated Cu(II) ion (0.413 cm^3 K mol^{-1} for g = 2.1) and magnetization (M) can be adjusted to a Brillouin law with S = ½ and g = 2.1, which shows that there is no appreciable interaction between the Cu(II) atoms.

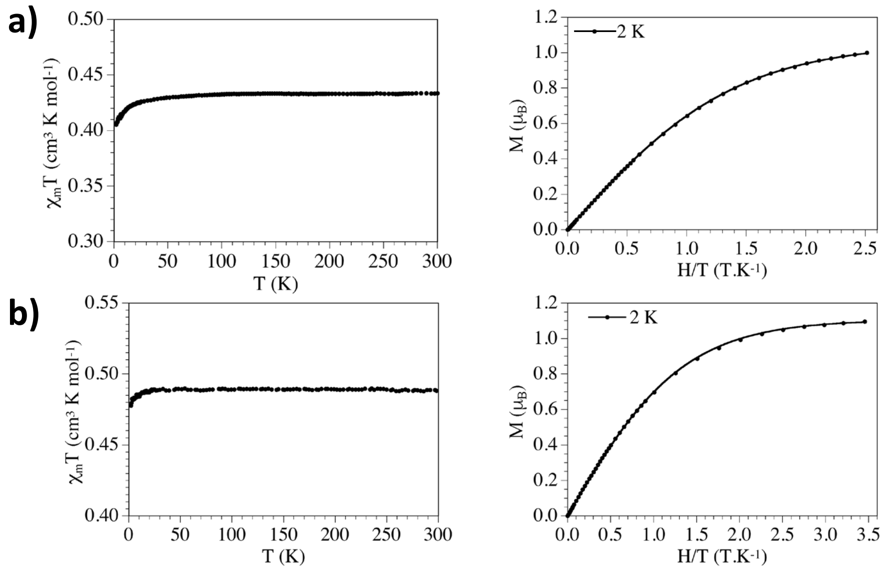

Figure S7. Thermal variation of $\chi_m T$ (left) and M (right) for compounds CuUPrO (**a**) and CuUPrO (**b**).

S2.3. Thermogravimetric Analysis

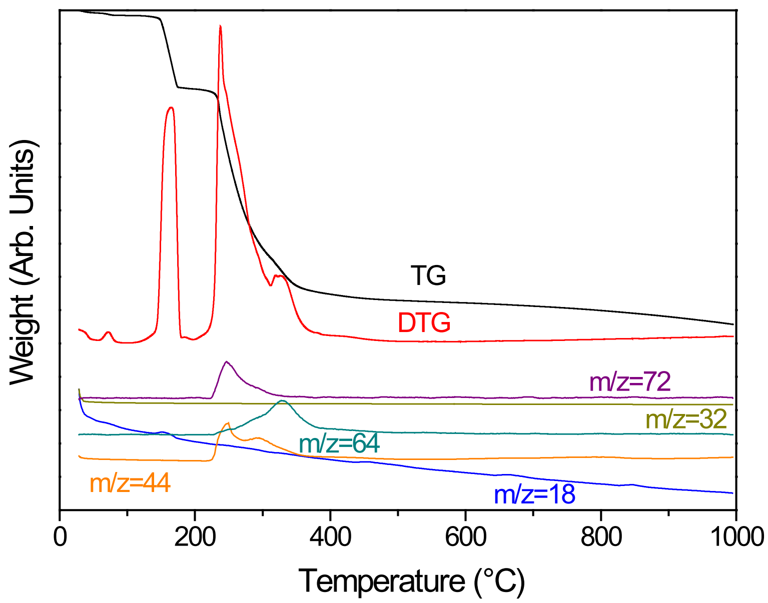

Figure S8. Thermal stability of compound CuUPrO in bulk, synthesized from $CuSO_4$ Thermogram signals: black and red line (weight (TG) and derivate weight (DTG), respectively) represent the stages of the losses produced. Multicolor-labelled lines represent the ion current associated with each mass lost in each stage.

The thermal study (Figure S8) shows a first stage around 200 °C probably indicative of the loss of one coordination water and one 4,4′-bipy molecules (obsd. 23.6%, calcd. 22.9 %). Next stages, from 200 to 1000 °C, imply the loss of two $UPrO^-$ molecules and the other 4,4′-bipy residue. (obsd. 70.6%, calcd. 69.0%). These molecules are mainly lost as CO_2 (m/z = 44) and there is clearly evidence about the presence of SO_2 (m/z = 64) regarding the thermogram below.

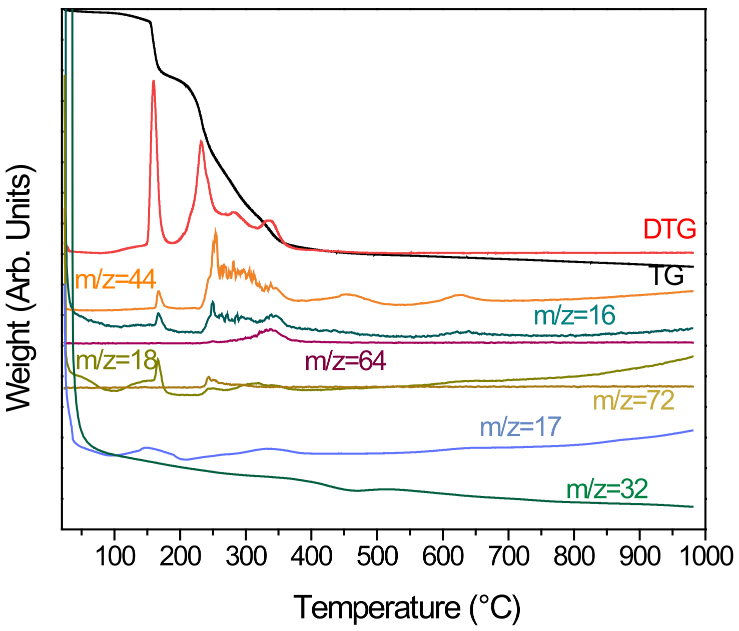

Figure S9. Thermal stability of compound CuUPrO@MOA**.** Thermogram signals: black and red line (weight (TG) and derivate weight (DTG), respectively) represent the stages of the losses produced. Multicolor-labelled lines represent the ion current associated with each mass lost in each stage.

This thermal study (Figure S9) shows a first stage at 188 °C probably indicative of the loss of methanol solvation, water molecule and one 4,4′-bipy molecules (obsd. 22.4%, calcd. 22.0%). Next stages, from 188 to 1000 °C, imply the loss of two $UPrO^-$ molecules and the other 4,4′-bipy residue. (obsd. 61.8%, calcd. 61.15%). As in the case of thermal study before, there is evidence about the presence of SO_2 (m/z=64) belonging to the sulphate groups.

S3. Morphological Study and EDX Analysis of CuUPrO and CuUPrO@MOA

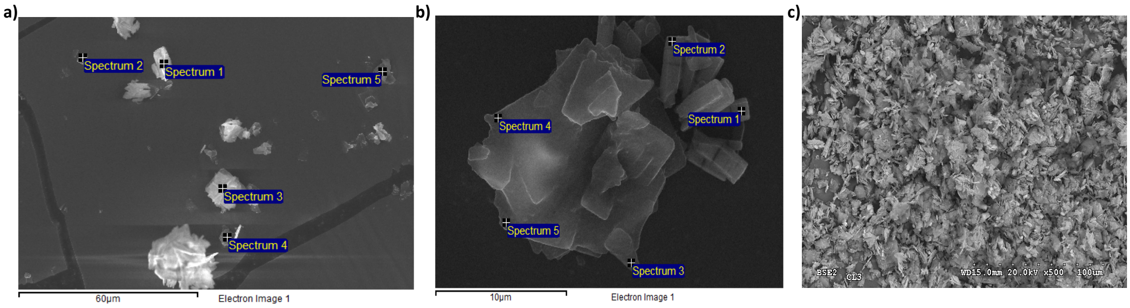

Figure S10. SEM-EDX images (**a**–**c**) for compound CuUPrO in bulk, synthesized from $CuSO_4 \cdot 5H_2O$.

Table S3. Energy-dispersive X-ray analysis for compound CuUPrO in which a corresponds to analysis in Figure S10a, b corresponds to FigureS10 b and c corresponds to Figure S10c. All results are in atomic %.

	Spectrum	C	N	O	Cu	S
**Fig. S10,a**	Spectrum 1	56.58	17.26	23.79	1.75	
	Spectrum 2	69.51	10.16	18.79	0.99	
	Spectrum 3	58.40	19.64	19.45	2.00	
	Spectrum 4	66.53	11.55	20.30	1.29	
	Spectrum 5	61.32	14.40	22.33	1.48	
**Fig. S10,b**	Spectrum 1	47.31	7.66	33.99	2.90	1.45
	Spectrum 2	49.97	8.99	31.75	2.69	1.21
	Spectrum 3	41.10		33.89	0.46	
	Spectrum 4	42.64	7.03	33.42	0.45	
	Spectrum 5	51.44	10.36	20.27	0.75	
	**Element**			**Mass. Norm (%)**		
**Fig. S10,c**	C			62.85		
	N			15.89		
	O			19.88		
	S			0.18		
	Cu			1.17		

The SEM-EDX analysis (Figure S10 and Table S3) showed the polycrystalline sample of compound CuUPrO contains traces of other phase (prismatic crystals in Figure S11, b, spectrum 1 and 2) as impurity that is reflected in Sulphur atomic % (Table S3, b) and in the XRD pattern (see Figure S4). Its distribution is not homogenous in the solid as we can see in Figure S10c where a large region of the same sample was analyzed.

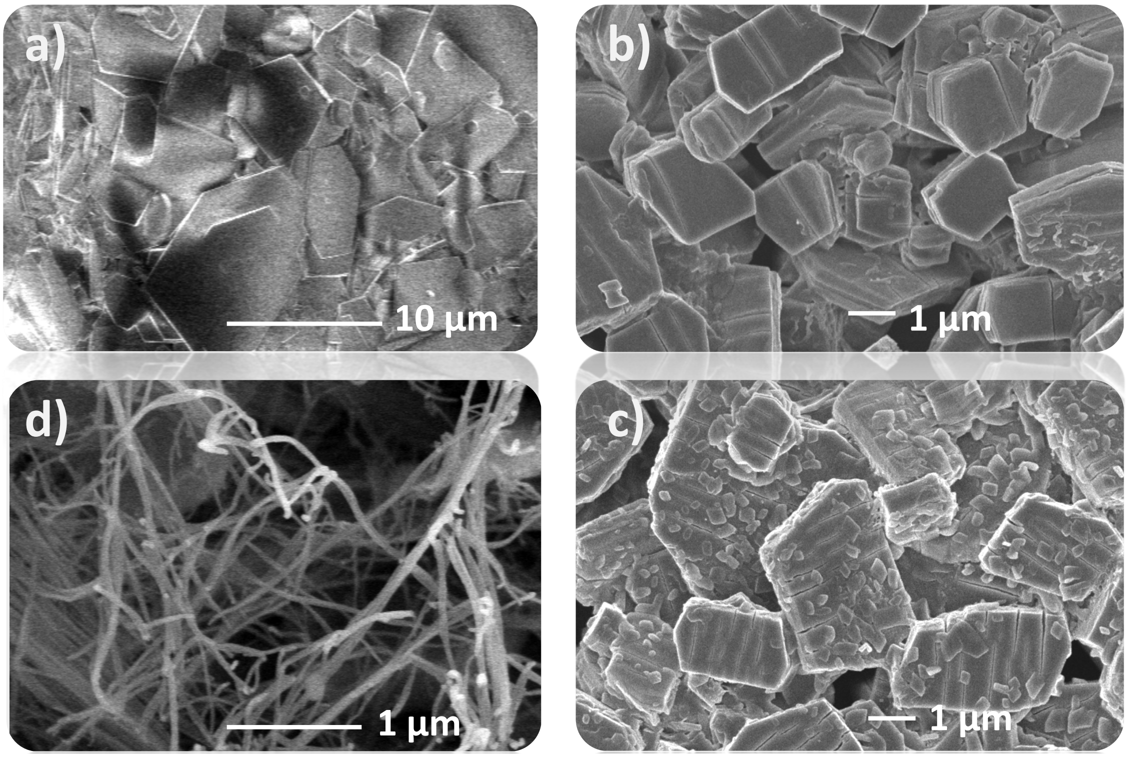

Figure S11. FESEM images of polycrystalline compound CuUPrO (**a**) and its transformation during sonication process (**b**), after 45 °C and its exchange with acetone (**c**) and finally, after its drying with supercritical CO_2 (**d**).

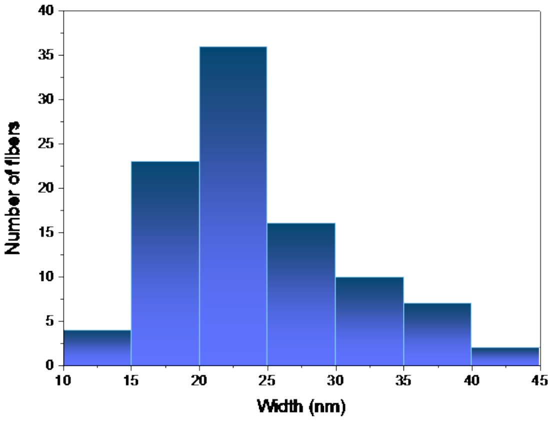

Figure S12. Statistical dimension based on the diameter of CuUPrO@MOA nanofibers.

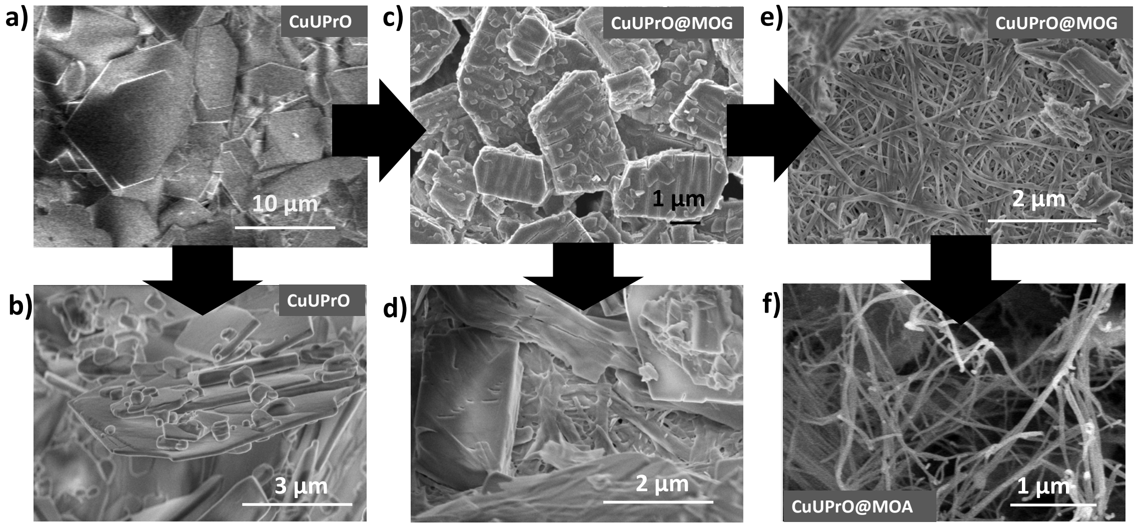

Figure S13. Schematic illustration of the experiments performed on pristine CuUPrO (**a**); after being subjected to Ar(g) for 5h at 50 °C (**b**) and after applying the gel method and exchanging with acetone to obtain CuPrO@MOG (**c**). Upon this treatment, CuPrO@MOG is going through Ar(g) for 5 h at 50 °C (**d**), liquid CO_2 for 5 h at 50 bar and 10 °C (**e**) and finally, when liquid CO_2 goes to supercritical state (40 °C, 80 bar) (**f**).

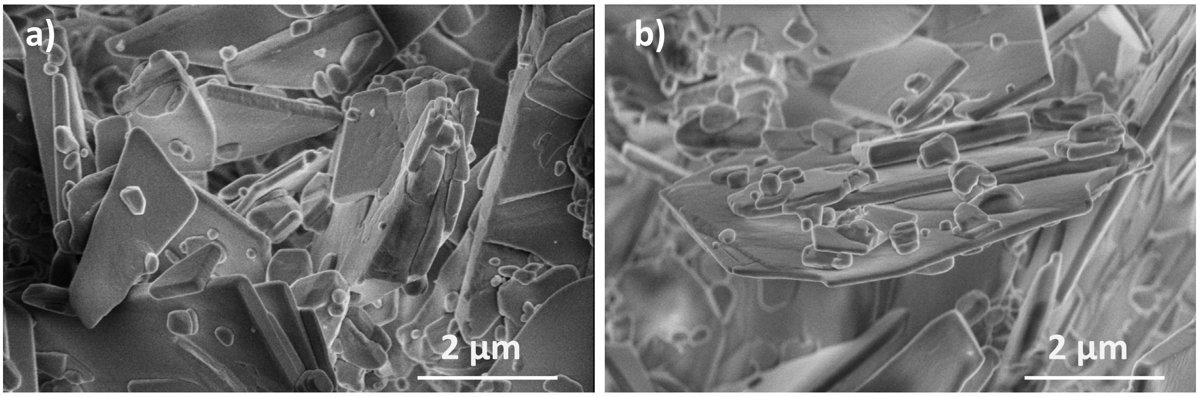

Figure S14. FESEM images of CuUPrO dispersed in methanol and subjected to liquid CO_2 for 5 h at 50 bar and 10 °C (**a**) and under Argon for 5 h at 50 bar at room temperature (**b**).

S4. Gel Formation Conditions

Table S4. Conditions of gel formation for 0.08 g of compound CuUPrO and 4 ml of solvent. UG: unconsolidated Gel; G: gel; C: colloidal suspension; P: precipitate; S: solution.

		Solvents		
**Temperature**	**Time**	**MeOH**	**EtOH**	**2-PrOH**
25ºC	24 h	UG	UG	C
	2 d	UG	UG	C
	7 d	UG	UG	C
45ºC	20'	UG	UG	C
	1 h	UG	UG	C
	4 h	G	G	P
70ºC	20'	UG	UG	C
	1 h	P	P	P

		4 h	P	P	P

Table S5. Stability of CuUPrO@MOG versus different organic solvents, temperature and acetic acid amount. UG: unconsolidated Gel, marked in yellow; G_v: violet gel, marked in violet; G_b: blue gel, marked in blue; C: colloidal suspension; P: precipitate; S: solution.

V_{AcOH}	Temperature	Time	Solvents		
			MeOH	EtOH	2-PrOH
0.2 ml + 15' sonication	25 °C	24 h	UG	UG	C
		1 d	UG	UG	C
		7 d	UG	UG	C
	45 °C	20'	G	G	C
		1 h	UG	UG	P
		4 h	UG	UG	S
	70 °C	20'	C	C	C
		1 h	P	P	P
		4 h	P	P	S
0.5 ml + 15' sonication	25 °C	24 h	C	C	C
		1 d	C	C	C
		7 d	C	C	C
	45 °C	20'	UG	UG	C
		1 h	P	P	P
		4 h	P	P	S
	70 °C	20'	C	C	C
		1 h	P	P	P
		4 h	S	S	S
0.75 ml + 15' sonication	25 °C	24 h	P	C	C
		1 d	C	C	C
		7 d	C	C	C
	45 °C	20'	C	C	C
		1 h	P	P	P
		4 h	P	P	S
	70 °C	20'	C	C	C
		1 h	P	P	P
		4 h	S	S	S

S5. Adsorption Study

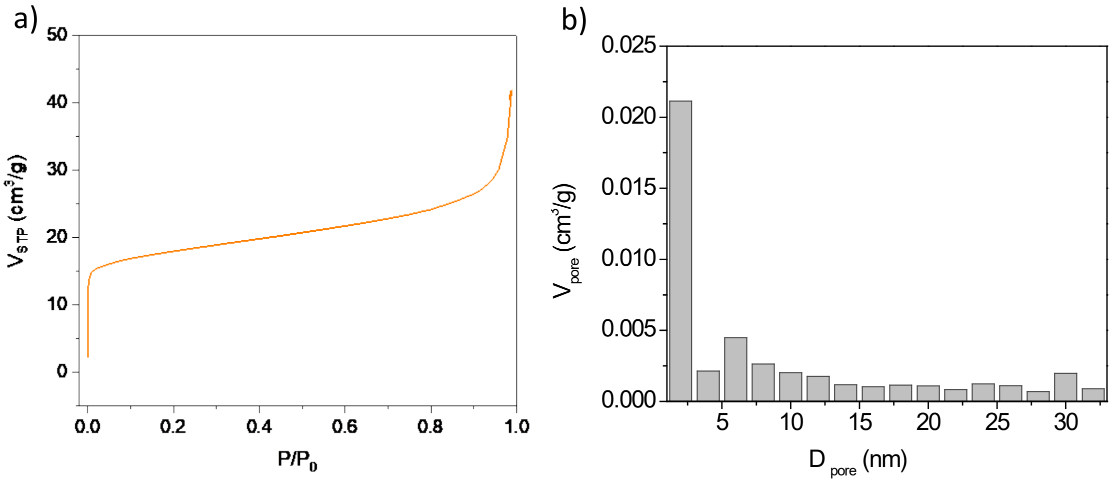

Figure S15. N_2 adsorption isotherms (77 K) (**a**) and pore size distribution histogram of CuUPrO@MOA (**b**).

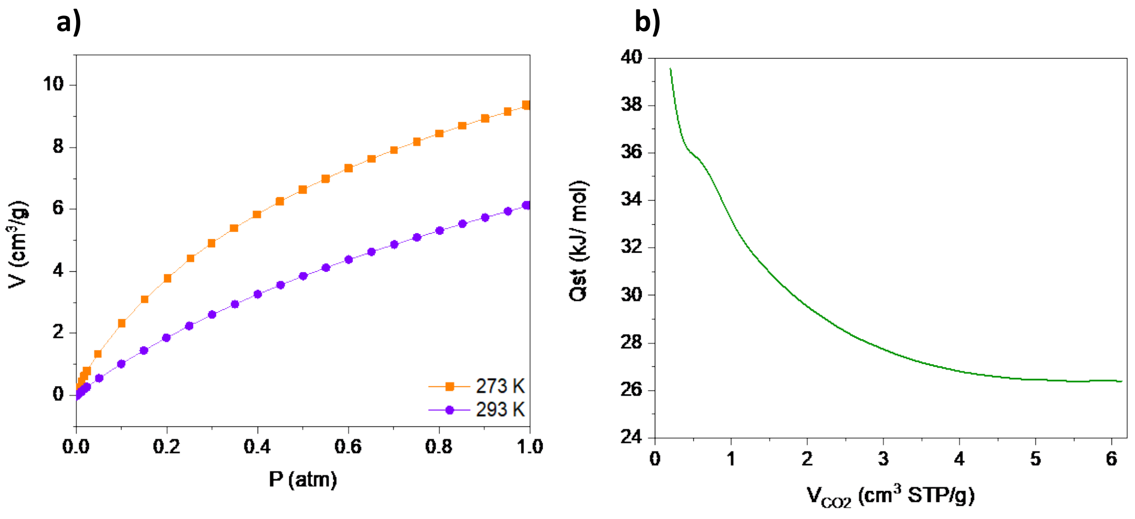

Figure S16. CO_2 adsorption isotherm of CuUPrO@MOA at 273 and 293 K (**a**) and isosteric heats vs CO_2 loading (**b**).

S6. Theoretical Methodology and Computational Details

All the ab initio calculations have been carried out by an adequate combination of the localized basis set Gaussian16 [41] and the plane-wave QUANTUM ESPRESSO atomistic simulation packages [42]. The Gaussian16 code has been employed, within the B3LYP/cc-pVTZ level of theory [52–54], to obtain optimized structures and characterize the interaction between the [Cu(UPrO)$_2$(4,4′-bipy)$_2$(H_2O)] discrete entities, with CO_2 and with different methylated nucleobases, as well as to analyze the effect in these interactions of a water solvation field. Solvent reaction field has been considered herein by the polarizable continuum model (PCM)[55] using the integral equation formalism variant, which creates the solute cavity via a set of overlapping spheres. On the other hand, the QUANTUM ESPRESSO package [42] has been adopted to account periodic conditions to evaluate intermolecular interactions within the crystalline structure of the compound. For this atomistic simulation code, one-electron wave functions are expanded on the basis of plane waves with an energy cutoff of 500 eV for kinetic energy. Electronic exchange and correlation (XC) effects have been accounted for by the revised generalized gradient corrected approximation of Perdew, Burke and Ernzerhof (GGA-PBE) [56]. Rappe–Rabe–Kaxiras–

Joannopoulos (RRKJ) ultrasoft pseudopotentials have been used to model the ion–electron interaction within the involved atomic species [57]. Brillouin zones (BZ) have been sampled using optimal Monkhorst–Pack grids. We have considered the DFT+D3 [58] perturbative correction accounting for the effect of van der Waals (vdW) forces. An empirical vdW correction is added to conventional energy density functionals in order to account for the R^{-6} behavior of the dispersive forces between atoms. The Fermi level was smeared out using the Methfessel–Paxton approach [59,60] with a Gaussian width of 0.01 eV, and all energies were extrapolated to $T = 0$ K. Self-consistency in the electron density was converged to achieve precision in the total energy better than 10^{-6} eV. The force threshold imposed for the geometrical optimization has been adjusted to reach a maximum net force acting on any atom below 0.04 eV Å^{-1}.

S6.1 DFT-calculation of the CO_2 interaction with CuUPrO

In order to elucidate the most favorable configurations, and their corresponding adsorption energies, upon the adsorption of CO_2 on CuUPrO, we have investigated a CuUPrO unit interacting with a CO_2 molecule in the three a priori most reactive sites, which are shown in Figure S18 and labelled as A, B and C.

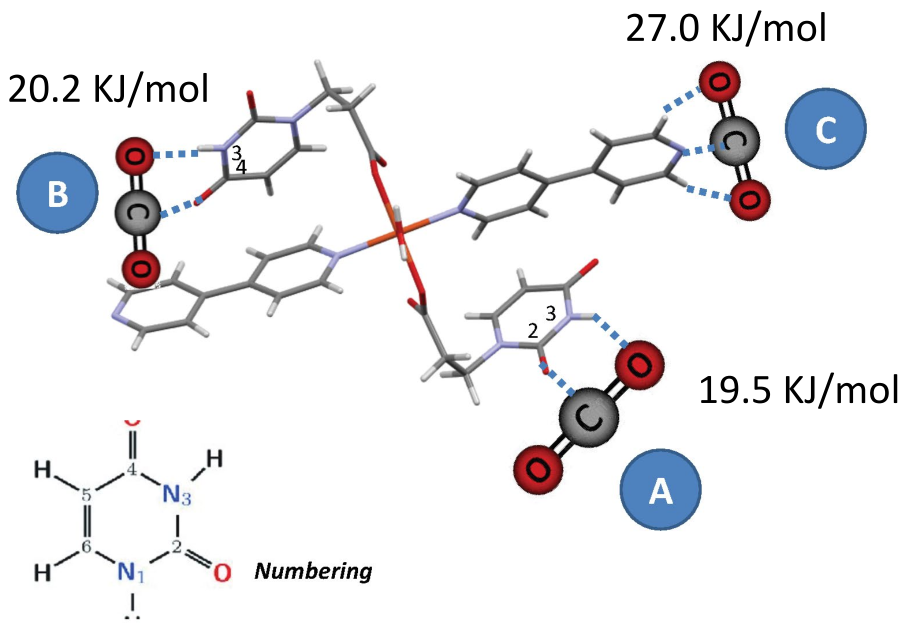

Figure S17. Pictorial sketch of the three most reactive sites upon the adsorption of CO_2 on a CuUPrO unit labelled as A, B and C.

S7. Separation of Nucleobases by HPLC: Stationary Phase Column and DFT Calculations

S7.1 Stationary Phase Column Optimization

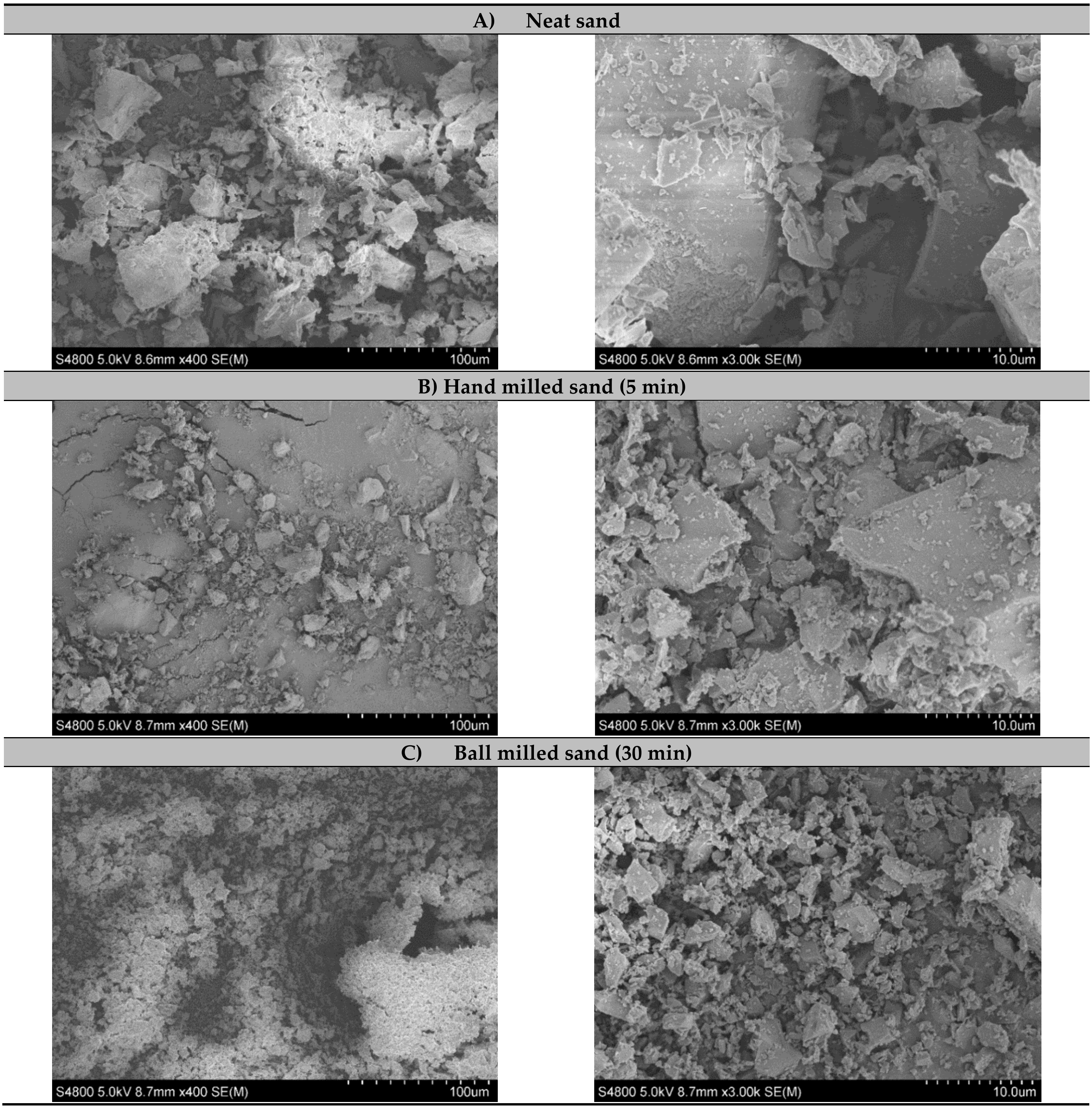

Figure S18. SEM images of the sample polydisperse in sizes. Grinding (especially the ball mill) reduces the maximum particle size, >40 µm for neat sand (**A**), 10–30 µm for hand milled sand (**B**), <5 µm for ball milled sand (**C**) and increases the fraction of smaller particles (<0.5 µm) and reduces qualitatively the polydispersion.

S7.2 DFT-Calculation of Adsorption Configurations and Interaction Energies of CuUPrO with the Methylated Nucleobases

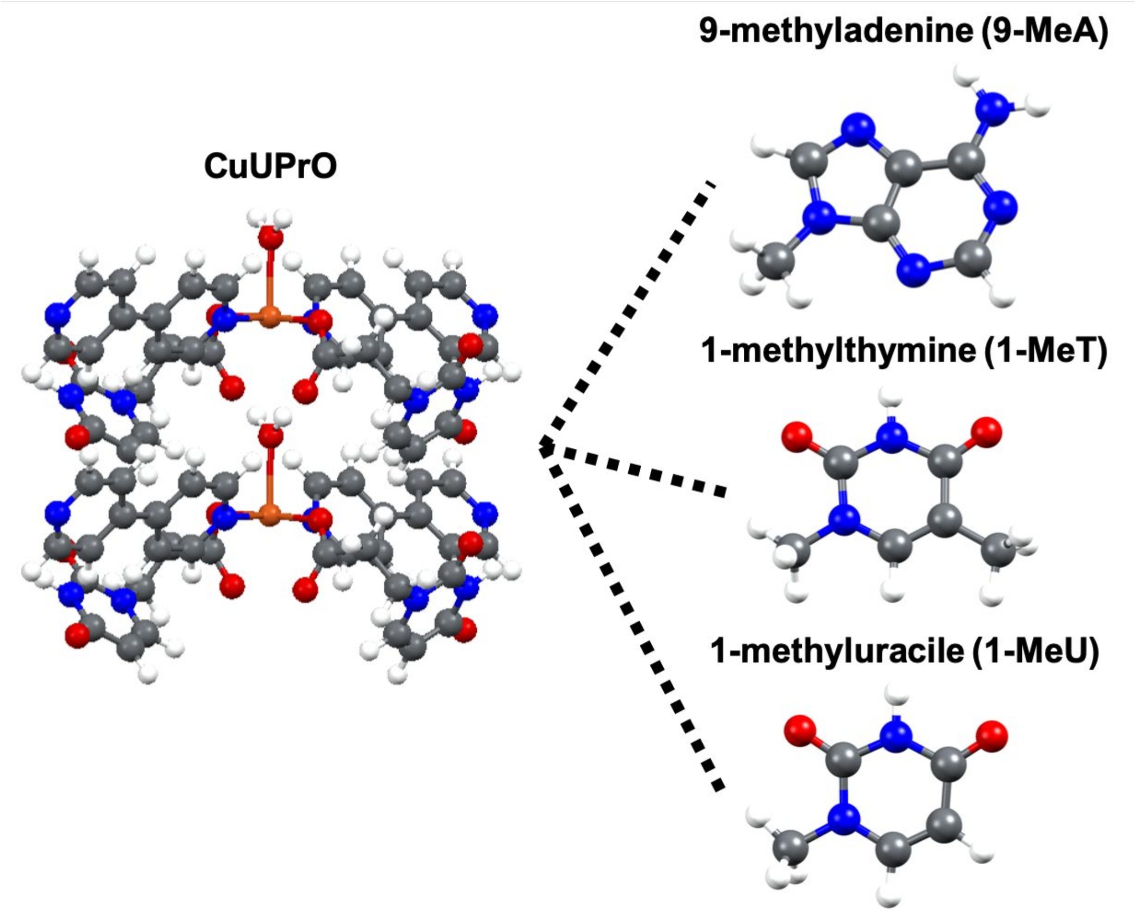

Figure S19. Pictorial sketch of a DFT-optimized two-entities model of CuUPrO to interact with the three methylated nucleobases 9-MeA, 1-MeT and 1-MeU (gas-phase structure).

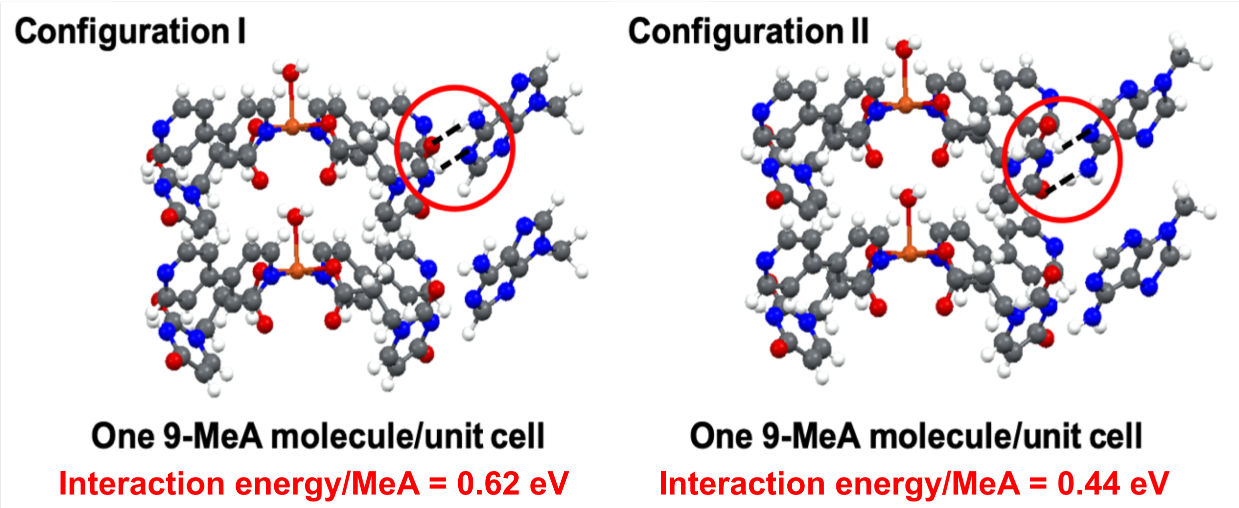

Figure S20. Different DFT-optimized configurations of 9-MeA adsorbed on the CuUPrO discrete entities with two different adsorption configurations. Two units are shown for a better appreciation

of the longitudinal "head-to-tail" monodimensional arrangement. Interaction energy per MeA is also indicated in each case.

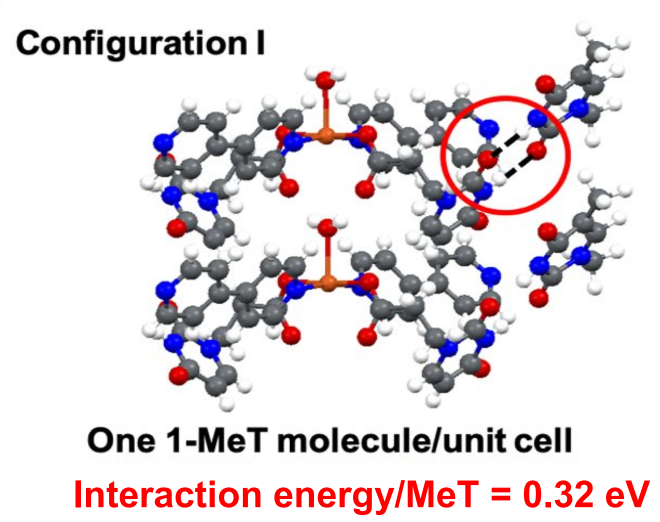

Figure S21. Different DFT-optimized configurations of 1-MeT adsorbed on the CuUPrO discrete entities with two different adsorption configurations. Two units are shown for a better appreciation of the longitudinal "head-to-tail" monodimensional arrangement. Interaction energy per 1-MeT is also indicated in each case.

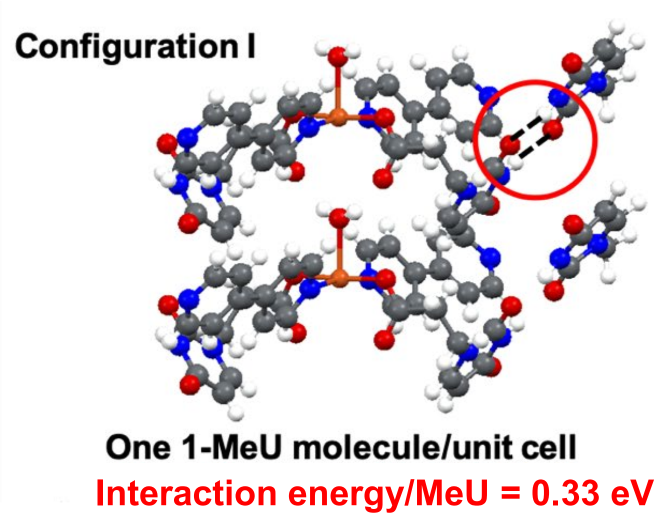

Figure S22. Different DFT-optimized configurations of 1-MeU adsorbed on the CuUPrO discrete entities with two different adsorption configurations. Two units are shown for a better appreciation of the longitudinal "head-to-tail" monodimensional arrangement. Interaction energy per 1-MeU is also indicated in each case.
